# Malnutrition Has No Effect on the Timing of Human Tooth Formation

**DOI:** 10.1371/journal.pone.0072274

**Published:** 2013-08-30

**Authors:** Fadil Elamin, Helen M. Liversidge

**Affiliations:** 1 Queen Mary University of London, Institute of Dentistry, Bart’s and The London School of Medicine and Dentistry, London, United Kingdom; 2 Khartoum Centre for Research and Medical Training, Qasr Street, Khartoum, Sudan; 3 El Razi Dental School, Elazhari 2, Khartoum, Sudan; Aga Khan University, Pakistan

## Abstract

The effect of nutrition on the timing of human tooth formation is poorly understood. Delays and advancements in dental maturation have all been reported as well as no effect. We investigated the effect of severe malnutrition on the timing of human tooth formation in a large representative sample of North Sudanese children. The sample (1102 males, 1013 females) consisted of stratified randomly selected healthy individuals in Khartoum, Sudan, aged 2-22 years using a cross-sectional design following the STROBE statement. Nutritional status was defined using WHO criteria of height and weight. Body mass index Z-scores and height for age Z-scores of ≤−2 (cut-off) were used to identify the malnourished group (N = 474) while the normal was defined by Z-scores of ≥0 (N = 799). Clinical and radiographic examination of individuals, with known ages of birth was performed including height and weight measurements. Mandibular left permanent teeth were assessed using eight crown and seven root established tooth formation stages. Mean age at *entry* and mean age *within* tooth stages were calculated for each available tooth stage in each group and compared using a t-test. Results show the mean age at entry and mean age within tooth stages were not significantly different between groups affected by severe malnutrition and normal children (p>0.05). This remarkable finding was evident across the span of dental development. We demonstrate that there is little measurable effect of sustained malnutrition on the average timing of tooth formation. This noteworthy finding supports the notion that teeth have substantial biological stability and are insulated from extreme nutritional conditions compared to other maturing body systems.

## Introduction

Dental development is integral in sustaining and maintaining the quality of life in humans. Nutritional deprivation is known to affect most body systems in the growing child [1]. The effect of malnutrition on developing teeth is not clear but there is evidence that dental maturity is insulated from environmental insults, such as severe malnutrition, compared to the skeleton and other body systems [2]. Human teeth develop as individual units and follow a sequence over a long period of time (over 20 years) during which they fully erupt into the mouth. Measuring tooth growth in living humans can be done by assessing their formation (crown and root stages), maturity (assessing overall tooth formation) and/or eruption (counting the number of teeth in the mouth) in relation to chronological age. There is considerable normal variation in age between individuals for the same tooth stage [3].

Malnutrition is thought to have a greater negative impact on skeletal development than growing teeth. This finding was observed in a study of 71 disadvantaged adopted young children, mostly under 4 years, measured on arrival and one year later [4]. Similarly, dental development in modern skeletal remains from Portugal (N = 41; 0–12 years) was shown to be less affected by socioeconomic status than skeletal growth and maturation [5]. A high correlation was found between both root stages of the canine tooth and skeletal maturity (medium phalanx of the third finger ) regardless of nutritional status in a study of stunted and nourished groups of children [6]. Animal studies consistently show a greater effect of malnutrition on mandibular growth compared to developing teeth but no clear association between tooth formation or eruption and malnutrition [7], [8].

The effect of nutrition on tooth formation and eruption has been investigated in a variety of ways which do not lend themselves to being compared. Past studies quantifying the effect of malnutrition on the timing of tooth formation have led to inconsistent and conflicting conclusions. Several methods investigating tooth formation and/or eruption in relation to malnutrition measured dental maturity as a single value on small samples over an age range making subgroup sample numbers very small. Interpreting these findings is difficult due to at least three reasons that relate to sampling. These include the wide age range during which teeth form (prior to birth up to early adulthood), the large age variation of developmental events and the lack of representative samples encompassing all developing teeth from crown to root apex.

Several methods exist to study the relationship between environmental factors and growing teeth. Development of the crown and root can be seen from radiographs and the ages of individual tooth stages can be compared between groups. The developing dentition in its entirety can also be viewed as a single dental maturity score for age [9]. The latter method is designed to assess maturity of an individual child but has been frequently used to compare dental maturity between groups. During root growth, teeth erupt into the mouth and the number of erupted teeth for age can be compared between groups.

Interpreting these findings is difficult due to a number of reasons including the wide age range during which teeth form (prior to birth up to early adulthood) and the large age variation of developmental events. Another problem is the lack of representative samples encompassing all developing teeth from crown to root apex. Many studies assess children over a wide age range resulting in small numbers in the groups being compared. This means that subgroups (normal and malnourished), age groups (younger and older) and tooth stages (early and late) are not evenly represented and findings may be influenced by the normal variation. Differences in such measures in small samples can be misleading if they fail to distinguish between findings that are the result of features of the age structure or sample size rather than the effect of nutrition.

Only 3 studies report the relationship between malnutrition and dental maturity and these are expressed as a single dental score. No link or delay between dental maturity and body mass index Z-scores (BMIZ) or stunting has been reported in 6–14 years in Brazil [10], 10 to 16 year olds in Peru [11] and 3–13 year olds in Iran [12].

The results from studies investigating malnutrition and tooth eruption are less clear with some showing no effect and some observing delay. With few exceptions, many studies fail to report the considerable variation in age and the number of teeth in the mouth. For example at 1 year of age a child can have between 2 to 10 deciduous erupted teeth while a girl aged 8.5 years can have between 9 to 20 permanent [13], [14]. Similarities between timing of tooth eruption in 324 stunted highland and lowland Ethiopian compared to United States children were reported in a sample aged 5–19 years [15]. A normal sequence of eruption of permanent teeth was observed in Nepal despite lifelong dietary deficiencies [16]. Malnutrition during the first five years of life was reported to have little effect on the number of later erupting permanent teeth for age [17]. A delay in eruption of the deciduous teeth was reported in stunted and wasted children compared to a normal group at each age [18]. A gradient was observed in the severity of malnutrition in relation to the number of teeth present; i.e. as malnutrition increased in severity, delays in eruption were observed, however, the age distribution of the stunted and wasted groups was small and uneven, with approximately half the age groups consisting of 5 or fewer subjects. An overall delay in eruption of both dentitions in groups defined by their BMIZ from Himachal Pradesh, India was reported [19], [20]. One study reports similar number of early erupting permanent teeth in wasted or stunted malnourished six year olds in Peru [18], [21], with more teeth present in the very small wasted and stunted group.

Overweight and obese children are thought to be advanced in both dental formation and the number of erupted teeth compared to children of normal weight. Heavier children showed a slight advancement in tooth formation across all ages evidenced by negative correlation of age of tooth stage and body fat [2]. Accelerated dental maturity was observed in obese or overweight children compared to normal and underweight children [22], [23] but subgroups consisted of only15 or 16 over a wide age range. Accelerated dental maturity was reported in 23 overweight and 18 obese children aged 8 to 15 [24]. Overweight children were reported to have more erupted teeth for age than children of normal weight [2], [25], [26], but variation is seldom detailed.

The aim of our study was to investigate the effect of malnutrition on the timing of tooth formation in humans from a structured sample of wide age range using well defined criteria.

## Materials and Methods

### Setting

The subjects (2–22 yrs, N = 2115) for this investigation were part of a population survey following the strengthening and the reporting of observational studies in epidemiology (STROBE) [27] that considered tooth formation, anomalies and disease in Khartoum, Sudan [28]. Initial growth analysis revealed a high prevalence of both chronic and acute malnutrition providing the opportunity to undertake this investigation. The effect of malnutrition on the timing of tooth formation was assessed only in individuals of Arab ethnic origin from the North of Sudan (Jaali, Mahasi, Shaigi, Bedairi, Halfawi and Dongalawi groups). Prior to 2011 Sudan was the largest country in Africa with a history of political instability, famine and high child mortality. There is considerable level of internal displacement due to long term internal civil conflicts.

### Study Design and Data Collection

Subjects were randomly selected from pre-schools, Khalwas (religious schools), mainstream schools and universities between January 2007 and May 2012 and stratified by school, using a two stage probability proportional to size cluster sampling [29].

Schools, preschools, Khalwas and universities in the three localities (Bahri, Umdurman and Khartoum City) in Khartoum were chosen from a list of schools in these localities obtained from the Ministry of Education and sampled where safety permitted. Children were excluded if the date of birth was unknown or if they had craniofacial anomalies. Ethical approval was granted by the Ethics committee at El Razi Dental School prior to the study (01/11/2006). Verbal and written consent was obtained from individuals and from parents of minors.

A dental examination was carried out and where appropriate, a dental panoramic radiograph taken (Siemens, Germany, 1988). Weight was measured to the nearest 0·1 kg and standing height to the nearest centimeter. Anthropometric measurements were assessed with light clothes and no shoes using calibrated scales and stadiometer (Seca 217 and 870 respectively, Seca, Hamburg, Germany). Dental treatment was provided where appropriate at no cost at the Dental School.

### Nutrtional Assessment

Chronic malnutrition was defined according to World Health Organization (WHO) standards and references. Z-scores for body mass index (BMI) for age and height for age (HA) were calculated to define the groups using WHO statistical packages [30]. A cut-off of Z-score of ≤−2 for both BMI and HA was used to select children to define the groups.

### Staging of Teeth

Permanent mandibular left teeth were staged by the first author from the radiographs using the 14 crown and root stages after Moorrees et al. (1963) in addition to staging the crypt of the third molar [31]. The radiographs were digitized, decoded and randomized for blind scoring by a person other than the investigator. Age of each subject was converted to decimal age. Intra-examiner reliability of stage assessment from 90 panoramic radiographs was assessed by Cohen’s Kappa with excellent agreement (K = 0.91).

### Statistical Analysis

SPSS, Release Version 17.0 (©SPSS, Inc., 2009, Chicago IL) was used to analyze data.

Intra-class correlation coefficients showed consistent height and weight measurements. Mean age of attainment for tooth stages in males and females was not different (p>0.05) and data were pooled [28].

## Results

**Figure 1:**
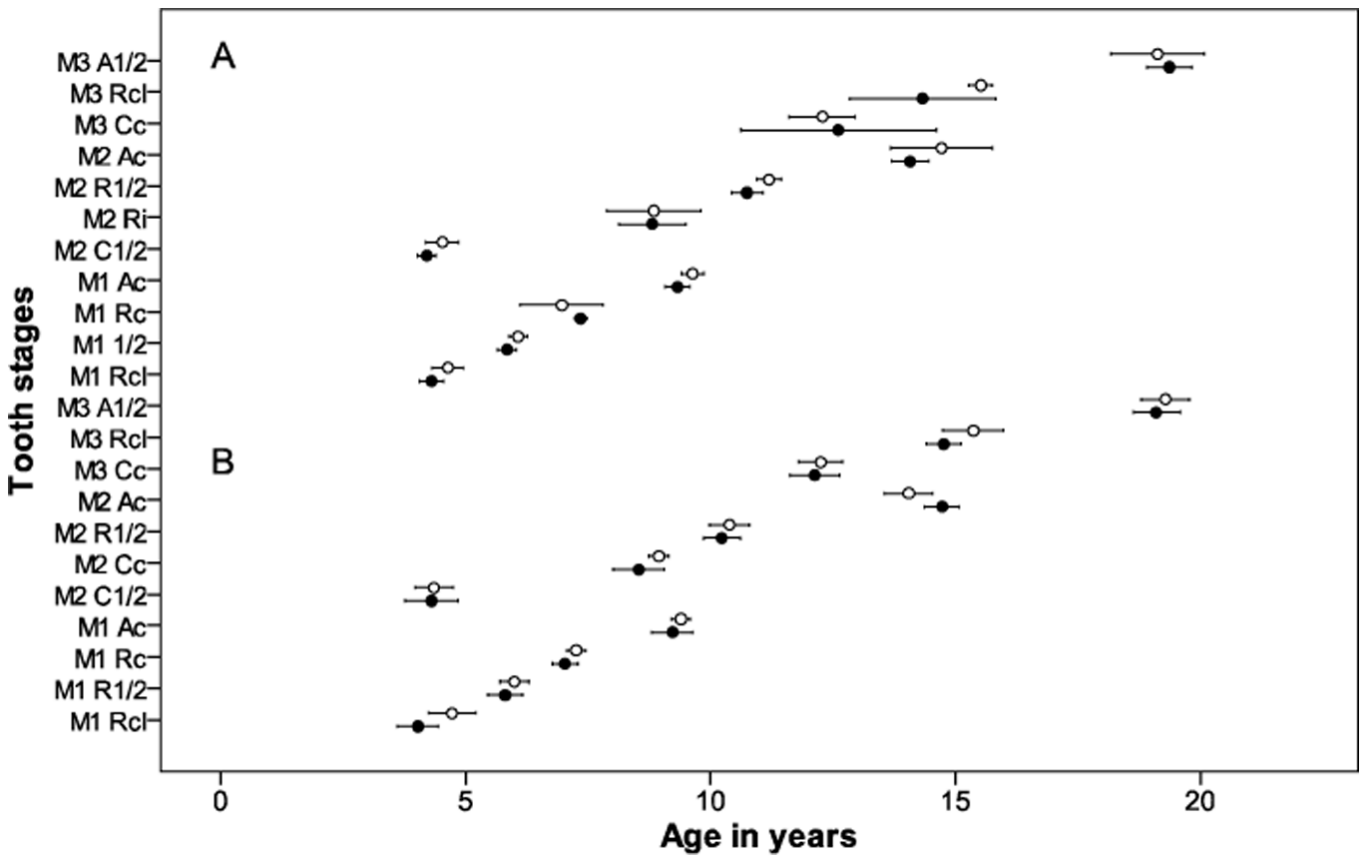
Mean ages of children entering tooth stages in malnourished and normal groups. Similarity of 95% confidence intervals of mean age *entering* selected tooth stages of molar teeth in malnourished and normal groups for body mass index (BMI) Z score shown in A and height for age (HA) Z score in B. This can be observed at each tooth stage. Open circles represent BMIZ and HAZ≤−2, dots represent BMIZ and HAZ ≥0. M1, first molar, M2, second molar, M3, third molar, C½, crown half formed, Cc, crown complete, Ri, initial root formation, Rcl, root cleft formation, R½, root half formed, Rc, root length complete, A½, apex half formed.

**Figure 2:**
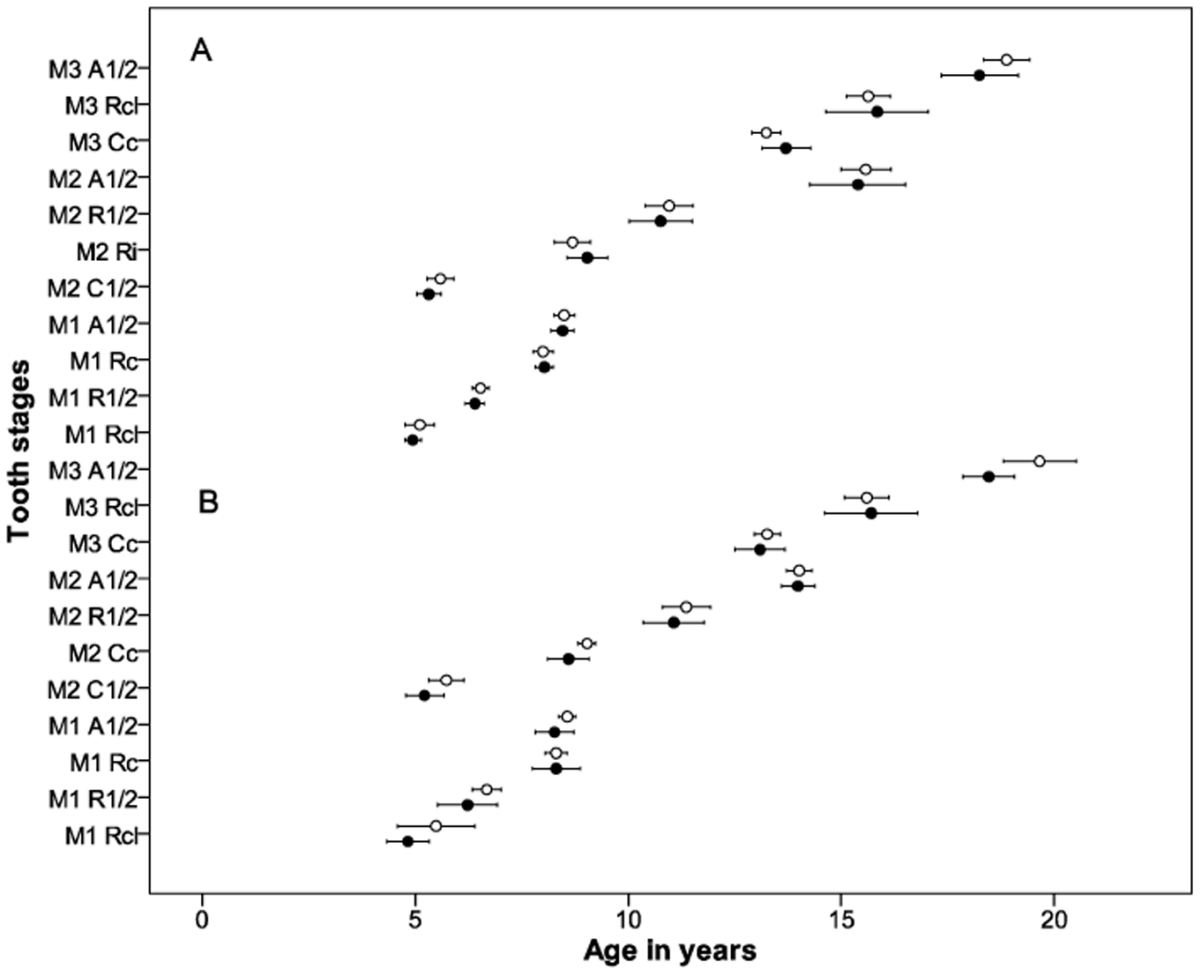
Mean age of children within tooth stages in malnourished and normal groups. Similarity of 95% confidence intervals of mean age *within* a selected tooth stages of molar teeth in malnourished and normal groups for body mass index (BMI) Z score shown in A and height for age (HA) Z score in B. This can be observed at each tooth stage. A and height for age (HA) Z score in B. This can be observed at each tooth stage. Open circles represent BMIZ and HAZ≤−2, dots represent BMIZ and HAZ ≥0. M1, first molar, M2, second molar, M3, third molar, C½, crown half formed, Cc, crown complete, Ri, initial root formation, Rcl, root cleft formation, R½, root half formed, Rc, root length complete, A½, apex half formed.

The age and sex distribution of the sample is shown in Table 1 while Tables 2 and 3 summarize the results for some molar tooth stages. The large number of tooth stages reduces the children per stage despite the large sample size due to the wide age range (Table 1). Two different statistical approaches were used to calculate mean age (mean age at *entry* and mean age *within* a stage). Probit regression with one year age groups was used in calculating the cumulative mean age at *entry* for tooth stages. Mean ages were compared between the normal and malnutrition group using student-tests (p<0.05). Both statistical approaches show that tooth formation for the group is similar in malnourished and normal groups (Figures 1 and 2).

**Table 1:** Age and sex distribution of study sample.

Age	N	N	BMIZ≥0	BMIZ≤−2	BMIZ≥0	BMIZ≤−2	HA≥0	HA≤−2	HA≥0	HA≤−2
	Female	Male	Female		Male		Female		Male	
2+	1	4	0	0	0	0	1	0	0	3
3+	31	35	5	4	10	6	16	10	31	7
4+	28	31	6	4	5	6	10	15	21	8
5+	29	44	6	5	9	5	14	12	30	11
6+	73	70	16	12	13	7	37	21	41	21
7+	79	80	13	24	39	7	54	10	52	23
8+	84	53	10	35	32	1	25	29	16	13
9+	77	68	15	39	48	4	31	28	23	33
10+	50	52	5	27	29	8	15	21	11	14
11+	45	62	8	25	36	10	14	19	10	25
12+	55	57	12	30	39	8	10	25	6	29
13+	38	72	8	23	43	14	11	11	6	42
14+	49	70	13	26	37	14	15	15	5	39
15+	51	63	15	25	41	10	13	24	8	40
16+	56	56	15	22	25	11	11	27	12	17
17+	71	67	19	17	22	21	15	34	17	31
18+	55	65	28	2	11	18	14	18	10	24
19+	33	48	19	2	11	14	14	7	10	22
20+	35	41	12	2	9	15	7	9	8	14
21+	34	39	20	1	2	19	11	13	13	9
22+	41	31	21	4	8	10	10	10	12	4
Total	1015	1108	266	329	470	208	348	358	342	429

Number of females and males for age categories and body mass index z (BMIZ) score and height for age z (HAZ) score. Age 2+indicates all children aged 2.00 to 2.99 years etc.

**Table 2:** Descriptives of age of entry and age *within* tooth stages in years between groups defined by Body Mass Index Z score.

Tooth stage	BMI	Mean age	95% CI	difference	SE diff	95% CI	P value
*Mean age at entry*							
M1 Rcl	Z≥0	4.03	3.61, 4.45	0.70	0.559	−2.07, 0.67	0.257
	Z≤−2	4.73	4.25. 5.22				
M1 R½	Z≥0	5.81	5.45, 6.16	0.20	0.532	−1.42, 1.04	0.730
	Z≤−2	6.00	5.70, 6.30				
M1 Rc	Z≥0	7.03	6.77, 7.29	0.23	0.435	−1.26, 0.80	0.614
	Z≤−2	7.26	7.06, 7.46				
M1 Ac	Z≥0	9.23	8.81, 9.64	0.17	0.641	−1.58, 1.24	0.796
	Z≤−2	9.40	9.21, 9.59				
M2 C½	Z≥0	4.31	3.77, 4.84	0.06	0.637	−1.60, 1.51	0.940
	Z≤−2	4.36	3.97, 4.75				
M2 Cc	Z≥0	8.54	8.01, 9.06	0.41	0.817	−2.19, 1.37	0.625
	Z≤−2	8.95	8.74, 9.15				
M2 R½	Z≥0	10.23	9.86, 10.60	0.16	0.603	−1.36, 1.22	0.795
	Z≤−2	10.39	9.98, 10.80				
M2 Ac	Z≥0	14.73	14.38, 15.08	0.69	0.736	−0.91, 2.67	0.372
	Z≤−2	14.05	13.56, 14.53				
M3 Cc	Z≥0	12.13	11.62, 12.64	0.12	0.944	−2.11, 1.87	0.900
	Z≤−2	12.25	11.80, 12.70				
M3 Rcl	Z≥0	14.76	14.40, 15.12	0.36	0.805	−0.21, 1.38	0.662
	Z≤−2	15.37	14.74, 15.99				
M3 A½	Z≥0	19.10	18.63, 19.58	0.19	1.037	−2.40, 2.02	0.857
	Z≤−2	19.29	1880, 19.78				
*Mean age within*							
M1 Rcl	Z≥0	4.83	4.34, 5.33	0.66	0.48	−1.68, 0.36	0.189
	Z≤−2	5.49	4.59, 6.40				
M1 R½	Z≥0	6.23	5.52, 6.93	0.46	0.38	−1.23, 0.32	0.236
	Z≤−2	6.68	6.34, 7.02				
M1 Rc	Z≥0	8.31	7.75, 8.87	0.01	0.28	−0.54, 0.56	0.980
	Z≤−2	8.31	8.05, 8.56				
M1 A½	Z≥0	8.27	7.82, 8.72	0.30	0.23	−0.76, 0.17	0.206
	Z≤−2	8.57	8.37, 8.76				
M2 C½	Z≥0	5.22	4.78, 5.67	0.51	0.33	−1.18, 0.17	0.135
	Z≤−2	5.73	5.31, 6.15				
M2 Cc	Z≥0	8.60	8.10, 9.09	0.43	0.29	−1.01, 0.15	0.140
	Z≤−2	9.03	8.83, 9.23				
M2 R½	Z≥0	11.07	10.35, 11.78	0.29	0.46	−1.23, 0.65	0.532
	Z≤−2	11.36	10.80, 11.92				
M2 A½	Z≥0	13.98	13.60, 14.37	0.03	0.25	−0.53, 0.47	0.907
	Z≤−2	14.01	13.71, 14.31				
M3 Cc	Z≥0	13.09	12.50, 13.68	0.17	0.33	−0.83, 0.49	0.609
	Z≤−2	13.26	12.96, 13.56				
M3 Rcl	Z≥0	15.70	14.61, 16.80	0.10	0.55	−1.00, 1.21	0.850
	Z≤−2	15.60	15.08, 16.12				
M3 A½	Z≥0	18.46	17.85, 19.07	1.19	0.60	−2.40, 0.01	0.052
	Z≤−2	19.65	18.80, 20.51				

The mean ages for tooth stages for groups defined by body mass index Z score (BMIZ) are similar and the difference between them is not significantly different to zero. This was observed at all molar stages for both statistical approaches.

95% CI 95% confidence interval, difference between normal and malnourished mean ages, SE diff standard error of mean difference, p value for 95% CI of difference in mean ages.

**Table 3:** Descriptives of age of entry and age *within* tooth stages (years) between groups defined by Height for Age Z score.

Tooth stage	HA	Mean age	95% CI	difference	SE diff	95% CI	P value
*Mean age at entry*							
M1 Rcl	Z≥0	4.31	4.06, 4.55	0.33	0.476	−1.42, 0.76	0.508
	Z≤−2	4.64	4.32, 4.95				
M1 R½	Z≥0	5.85	5.65, 6.04	0.22	0.485	−1.34, 0.90	0.662
	Z≤−2	6.07	5.89, 6.26				
M1 Rc	Z≥0	7.35	7.22, 7.48	0.38	0.522	−0.82, 1.56	0.488
	Z≤−2	6.97	6.12, 7.81				
M1 Ac	Z≥0	9.33	9.08, 9.57	0.31	0.595	−1.64, 1.02	0.613
	Z≤−2	9.64	9.41, 9.86				
M2 C½	Z≥0	4.21	4.02, 4.40	0.32	0.545	−1.61, 0.97	0.576
	Z≤−2	4.53	4.19, 4.86				
M2 Ri	Z≥0	8.81	8.13, 9.49	0.04	0.834	−1.84, 1.76	0.963
	Z≤−2	8.85	7.89, 9.81				
M2 R½	Z≥0	10.75	10.43, 11.08	0.45	0.727	−2.05, 1.15	0.549
	Z≤−2	11.20	10.95, 11.45				
M2 Ac	Z≥0	14.08	13.70, 14.46	0.64	0.815	−2.46, 1.18	0.450
	Z≤−2	14.72	13.68, 15.75				
M3 Cc	Z≥0	12.61	10.62, 14.61	0.32	0.904	−1.67, 2.31	0.703
	Z≤−2	12.29	11.61, 12.96				
M3 Rcl	Z≥0	14.33	12.84, 15.82	1.19	0.935	−3.23, 0.85	0.227
	Z≤−2	15.52	15.27, 15.76				
M3 A½	Z≥0	19.37	18.91, 19.83	0.24	0.960	−1.82, 2.30	0.806
	Z≤−2	19.13	18.17, 20.09				
*Mean age within*							
M1 Rcl	Z≥0	4.94	4.76, 5.13	0.16	0.180	−0.52, 0.20	0.377
	Z≤−2	5.11	4.77, 5.44				
M1 R½	Z≥0	6.40	6.18, 6.62	0.13	0.166	−0.46, 0.21	0.442
	Z≤−2	6.53	6.34, 6.72				
M1 Rc	Z≥0	8.03	7.82, 8.24	0.03	0.159	−0.28, 0.35	0.835
	Z≤−2	8.00	7.76, 8.23				
M1 A½	Z≥0	8.46	8.19, 8.72	0.04	0.187	−0.41, 0.33	0.837
	Z≤−2	8.49	8.25, 8.74				
M2 C½	Z≥0	5.32	5.04, 5.61	0.27	0.218	−0.70, 0.16	0.220
	Z≤−2	5.59	5.29, 5.90				
M2 Ri	Z≥0	9.04	8.56, 9.52	0.35	0.326	−0.31, 1.00	0.293
	Z≤−2	8.69	8.27, 9.12				
M2 R½	Z≥0	10.76	10.03, 11.50	0.19	0.477	−1.16, 0.77	0.686
	Z≤−2	10.96	10.40, 11.52				
M2 A½	Z≥0	15.39	14.26, 16.51	0.19	0.586	−1.35, 0.98	0.751
	Z≤−2	15.57	14.99, 16.16				
M3 Cc	Z≥0	13.70	13.14, 14.27	0.46	0.321	−0.17, 1.10	0.152
	Z≤−2	13.24	12.90, 13.58				
M3 Rcl	Z≥0	15.84	14.64, 17.04	0.21	0.565	−0.94, 1.36	0.711
	Z≤−2	15.63	15.12, 16.15				
M3 A½	Z≥0	18.24	17.34, 19.15	0.64	0.507	−1.66, 0.38	0.214
	Z≤−2	18.88	18.34, 19.42				

The mean ages for tooth stages for groups defined by height for age Z score (HAZ) are similar and the difference between them is not significantly different to zero. This was observed at all molar stages for both statistical approaches.

95% CI 95% confidence interval, difference between normal and malnourished mean ages, SE diff standard error of mean difference, p value for 95% CI of difference in mean ages.

The control group with Z-scores of ≥0 (normal) for BMI and HA were considered to exhibit normal growth (Table 1). In this study low HA Z-scores indicates long-term chronic malnutrition while low BMI Z-scores for age indicate current poor nutritional status in this population. The prevalence of stunted growth in this sample was 25%.

Mean ages at *entry* of molar stages were not significantly different (p>0.05) in the severely malnourished and control groups. This cumulative approach of analysis is robust and takes into account all the individuals within the sample who are yet to reach a specific tooth stage and those who have attained or passed the stage. A less robust but more reported method is to compare the mean age of individuals *within* a particular tooth stage. The sample size, usually limited by practical difficulties, in the latter approach is considerably smaller with cumulative statistics. Mean ages *within* molar tooth stages were not significantly different (p>0.05) between the severely malnourished and control groups as shown in Figure 2.

## Discussion

Changes in food habits are known to have an impact on the growth of children, states of health and disease in populations, overall life expectancy as well as having a direct impact on the quality of life. The investigation of dental development of children affected by acute and chronic malnutrition is difficult despite advances in child growth standards and references published by the World Health Organization in 2006 [30]. Our knowledge is drawn from studies of small samples using differing sampling techniques and methods making comparisons difficult. Some studies fail to detail the number of malnourished children, per age group and the three studies that report results use a method of dental maturity expressed as a single value that hides timing and age variation of individual tooth stages.

Eid et al. [10] investigated dental maturity using Demirjian’s method in nutritionally similar groups to those in our study. The age range of their sample was 6–14 years. The undernutrition group (below 5th percentile for BMI), consisted of 11 boys and 20 girls. The Normal group (between 15th to 85th percentile for BMI), consisted of 214 girls and 189 boys. The difference between dental and chronological ages between these groups was not significantly different (calculated as P = 0.526 girls, P = 0.823 boys). No information on the exact ages of the 11 boys and 20 malnourished girls was detailed. Bagharian and Sadeghi [12] compared normal (168 boys, 184 girls) and undernourished children (49 boys, 45 girls) below 5th percentile for BMI groups aged 3 to 13 years of age using the method of Demirjian. They report differences between dental and chronological ages as 0.15 year in malnourished and 0.17 year in normal children (not significantly different, calculated as P = 0.720). We were unable to compare individual tooth stages because Demirjian’s method expresses dental maturity as a single score.

Camerieri et al. [11] compared similar groups to those in our study. The age range of undernourished and normal children was between 9 and 16 years of age. Dental maturity scores were similar in undernourished and normal children for all age groups using two methods of dental maturity. This similarity between groups is in agreement with our findings. One of the dental maturity methods they used was Demirjian’s. For this particular age range, percentage dentally mature was 89.4% and 88.2% for undernourished and normal ten year olds respectively. In other words, the largest difference between the groups was around one percent. At the age range of this study sample, tooth development is limited to root formation in late forming permanent teeth; namely canines, premolars, second molars as well as third molar development. The two dental maturity methods they used exclude third molars. Our study considered individual stages with a much wider age range encompasses early, middle and late forming teeth including third molars.

Flores-Mir et al. (2005) noted a high correlation between both root stages of the canine tooth and skeletal maturity (medium phalanx of the third finger ) in both stunted and nourished groups of children aged 9 to 16 years. In order to measure the effect of nutrition on growth and development, teeth and bones have to be examined before they reach maturity. The tabulated results from Flores-Mir et al. (2005) show that 27% of their sample were skeletally mature and 32% were dentally mature (canine root apex mature). This illustrates the complexity of investigating growing teeth in relation to developing bones in a sample that is made up of almost a third mature children.

In our study, severe malnutrition (Z≤−2) had no measurable impact on the timing of tooth formation. Early and late forming molar stages of the first, second and third molar teeth (spanning dental formation time of all teeth) showed no difference in the timing of their formation between groups. This remarkable result shows that the timing of the developing dentition is unaffected by sustained severe environmental insults. The stratified structured sampling strategy enabled comparisons to be representative of the population. The distribution of children in our sample was spread across age groups from early age to completion of the third molar.This allowed us to examine the effects of malnutrition on individual tooth stages across the span of development rather than a single measure of dental maturity. Selecting tooth stages of the first, second and third molars cover much of the postnatal developing dentition. A clear cut-off criteria of ≤−2 Z scores was used to define malnutrition. The location of the study resulted in a considerable sample size for the malnourished group compared to previous studies investigating tooth formation. This region of Africa has long been affected by internal conflicts, famine and high burden of disease with more than 48% of children under 5 years stunted (WHO global data base on Child growth and Malnutrition;15 Sept 2010). The malnourished and normal groups in our study are similar in tooth formation timing despite having diverse genetic and linguistic heterogeneity known to exist between sub-Saharan groups [32]. Our choice of two different statistical approaches of age of *entry* and age *within* tooth stage overcomes misinterpreting the variability that is inherent to developing teeth when individuals or small groups are compared. Both methods demonstrate no significant differences in timings of early and later tooth formation stages between children with chronic malnutrition and groups defined as normal for this population.

This study has several limitations making direct comparison with other investigations difficult. Previous reports investigating the effect of malnutrition on the timing of the developing dentition use a number of methods and some fail to adequately report variation. Few of these compare average age of individual teeth in sufficiently large subgroups over a wide age range to allow environmental effects to be quantified. Despite our large sample size, some age and BMI Z-score categories and tooth stages contain small numbers. Additionally, our study sample did not contain children during the first few years of life. This is in common with most other studies using dental radiographs due to the practical and ethical difficulties. In order to examine the full spectrum of nutritional effects, it is important to assess the full spectrum of malnourished, overweight and obese children, however, our study was skewed towards under-nutrition. No reliable reference growth data are available from this region in Africa and this represents the first study of its kind as there are no published population references for tooth formation available for comparison. Furthermore, the effect of malnutrition on other important aspects remains unanswered and work is needed on tooth shape, size and structure, hypoplasia, eruption, exfoliation, jaw growth and skeletal maturation in these groups.

Severe malnutrition appears to have a minimal effect on the timing of tooth formation despite the large variation that exists between individuals. This study highlights the stability of the developing dentition in relation to other biological systems which are thought to be affected by malnutrition. Our results will be of interest to clinical disciplines including dental health care, orthodontics, nutrition, as well as the study of evolution, forensic science and anthropology. This will enable better estimates of dental maturity for age, better prediction of growth of the jaws and teeth and the improved timing of dental and orthodontic treatment.

Therefore, when factors such as wide age range, large representative sample size, statistical method of analysis, the use of established and defined growth and nutritional criteria are controlled for, our results show that the effect of extreme malnutrition on the timing of tooth formation was negligible. These findings emphasize the biological stability in the timing of the developing dentition in humans.

We conclude that the timing of developing teeth is less affected by extremes compared to other body systems.
